# How to handle and care for bulbs in ophthalmic equipment

**Published:** 2013

**Authors:** Ismael Cordero

**Affiliations:** Clinical engineer

**Figure:**
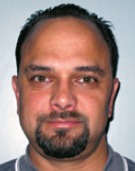
Ismael Cordero

Many devices used in eye care rely on light bulbs or lamps for their operation. All light bulbs have a limited lifespan and when the bulb fails the device becomes unusable. Therefore, knowing how to handle, how to inspect and how to replace bulbs is important. Just as important is keeping spare bulbs to hand!

## Prolonging the lifespan of bulbs

The lifespan of a bulb varies widely among different types of bulbs and also will depend on the particular application and the environment in which it is used, among many other factors. To help ensure the longest bulb life possible you should follow these guidelines.

- Turn on the equipment at the lowest light intensity setting. Sudden high voltage surges can blow bulbs, especially when cold.
- Use the equipment at lower light intensity settings as much as possible.
- Turn off the bulb or equipment when it is not being used.
- Don't move a device while the bulb is still lit or hot: wait until the bulb has cooled. Even gentle vibrations may cause a hot bulb filament to break since they are more brittle when hot.
- Each time you turn the unit on, a current surge stresses the bulb's filament. The more often this stress is applied the sooner the bulb will fail. For this reason, turning the equipment on and off frequently is not recommended.
- Inadquate cooling can cause the bulb envelope seal to fail or the bulb capsule to swell. Make sure that the fan, if included, is operating and that the intake and exhaust vents are not blocked. Keep the filters clean and the area around the equipment free from objects that might restrict airflow and create heat.
  **Figure 1.**
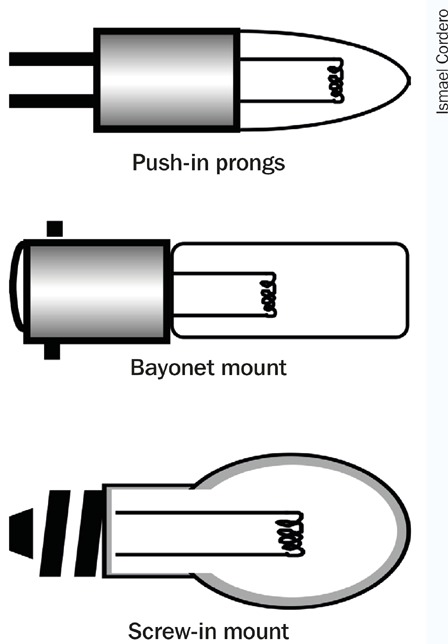
Different connection types

**Figure:**
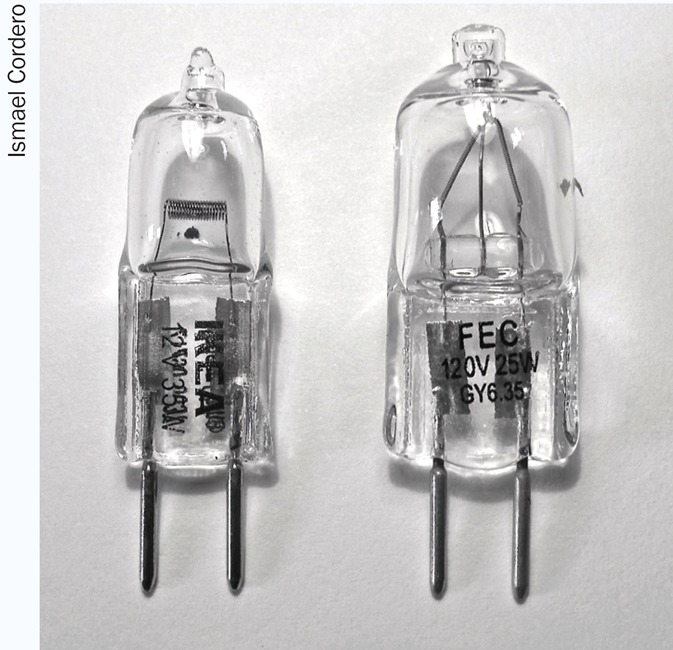
Similar but different bulbs
- High power line voltage is a major cause of short bulb life. Typically an increase of 5% in the voltage supply above the bulb's rated voltage can reduce its lifespan by 50%.
- Oilsandotherstainsonthe bulb's glass can create hot spots that can cause the bulb to fail. You should avoid touching bulbs with your bare fingers.
- Pitting, corrosion or other damage in the bulb socket's contacts will cause inconsistent current and will shorten the bulb's life. Replace damaged sockets.

## Removing and installing bulbs

- Shut off the instrument and unplug it from the electrical outlet.
- Let the bulb cool before removing it. You should remember that the bulb-especially if it is a halogen bulb-will be very hot and could burn your fingers. You should allow sufficient cooling time and use a cloth or a suitable heat insulator to hold the bulb.
- Do not touch the new bulb directly with your fingers; use tissue or cotton gloves. If the bulb is touched accidentally, it should be wiped clean with a cloth moistened in alcohol to remove potential skin oil deposits. These deposits can burn into the glass, creating shadows and weakening the glass, and causing premature failure.
- Know how each specific bulb fits into its socket (Figure 1).
- When installing bulbs, be sure the lamps are secured completely. The tendency is to stop at the first sign of resistance. Continue to carefully apply force at the base of the lamp until you are sure the lamp is secure. Improper installations will cause electrical arcing, overheating and shorten the lifespan of both lamp and socket.
- Check that the filament is correctly aligned to ensure that the light projected is of even intensity.
- Replace a defective bulb with the identical type (same shape, voltage and wattage). Some bulbs may look very similar but may have quite different heat characteristics that could cause damage or fire risk. The two bulbs in Figure 2 look alike and can both fit into the same type of socket, but one is a 12 volt/35 Watt bulb, while the other is a 120 volV25 Watt bulb. Also, the filaments have different shapes and will yield different light profiles.

## Inspecting bulbs

- Check for bent or sagging filaments as these indicate imminent failure.
- Inspect the filament for continuity and welding points. Loose filaments will produce a blue arc of light and flickering.
  **Figure 3.**
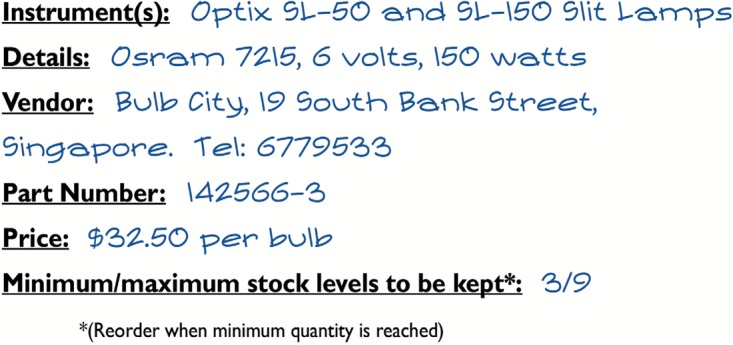
Bulb stock can be kept in a container and labelled as shown
- A metallic haze on the inside of the glass envelope of the bulb signifies evaporation of the filament, precedes filament failure, and also reduces the lamp's brightness.
- Inspect the bulb's contacts for corrosion. Sometimes it is possible to remove the corrosion with a file or sandpaper.

## Maintaining a bulb inventory

- Based on the number of instruments you have that require a specific type of bulb, and on how often you replace this type of bulb, you should purchase and store replacements in clearly labeled containers. Figure 3 shows one way to label the bulb containers.

## General handling and safety precautions

- Always turn off the electrical power before inserting, removing, or cleaning a bulb.
- Always handle bulbs with care and store them appropriately to minimise the likelihood of glass breakage. If you do break a bulb, please remember that some contain harmful substances and should be handled accordingly. Incandescent bulbs pose little or no threat except that of the broken glass and can be dealt with as regular waste. Fluorescent tubes and most discharge bulbs can contain potentially harmful chemicals that should be handled with care and disposed of in accordance with your local waste authority rules and health and safety policies.
- Bulbs should be easy to install and remove from their fittings and should never be forced as this can often result in breakage of the glass.
- Many bulbs contain gases at either greater than or less than atmospheric pressure and may either explode or implode if the glass is broken. This can cause a significant hazard. Bulbs should not be disposed of by breaking them unless appropriate protective equipment is used and environmentally sound disposal methods are followed.
- Do not use halogen or other hot burning bulbs near paper, cloth or other combustible materials that can catch fire.
- Do not look directly at an operating bulb for any period of time; this may cause serious eye injury.

